# Cardiac structure and inflammation drive memory impairment via dual pathways of heart-brain axis dysregulation in atrial fibrillation patients undergoing catheter ablation

**DOI:** 10.3389/fnins.2025.1725953

**Published:** 2025-11-27

**Authors:** Xin Chen, Jie Ni, Yu Wang, Dandan Kou, Danni Ge, Xunwen Xue, Yingming Zhao, Biao Xu, Ming Li, Jiu Chen

**Affiliations:** 1 Department of Cardiology, Nanjing Drum Tower Hospital, Nanjing Drum Tower Hospital Clinical College of Nanjing Medical University, Nanjing, China; 2 Department of Cardiology, Nanjing Drum Tower Hospital, Affiliated Hospital of Medical School, Nanjing University, Nanjing, China; 3 Department of General Medical, Nanjing Drum Tower Hospital, the Affiliated Hospital of Nanjing University Medical School, Nanjing, China; 4 Department of Radiology, Nanjing Drum Tower Hospital, Affiliated Hospital of Medical School, Nanjing University, Nanjing, China; 5 Institute of Medical Imaging and Artificial Intelligence, Nanjing University, Nanjing, China; 6 Medical Imaging Center, Nanjing Drum Tower Hospital, Affiliated Hospital of Nanjing University Medical School, Nanjing, China

**Keywords:** atrial fibrillation, catheter ablation, memory impairment, heart-brain axis, functional connectivity

## Abstract

**Background:**

Atrial fibrillation (AF) patients undergoing catheter ablation (CA) frequently present with cardiac structural and functional alterations and persistent memory impairment. This study aimed to investigate how cardiac structure and function impacts memory-related brain structure and function, whether CA reverses impaired memory networks, and to delineate the dual-pathway regulatory mechanism of the heart-brain axis underlying AF-associated memory deficits.

**Methods:**

Thirty-eight AF patients underwent longitudinal assessments [memory function tests, clinical evaluations including blood biomarkers and cardiac function, structural/functional magnetic resonance imaging (MRI)] before CA and on postoperative day 7. Forty-five healthy controls (CN) were included for comparison. Hippocampal functional connectivity (FC) and voxel-based morphometry were used to quantify memory-related brain networks and gray matter (GM) volume. Bivariate correlations explored relationships between GM alterations, hippocampal FC, memory performance, and clinical features (cardiac structural parameters and blood-borne inflammatory markers).

**Results:**

First, compared with CN, AF patients showed memory impairment, reduced GM volume in the bilateral calcarine cortex, cuneus, lingual gyrus, inferior/middle temporal gyri, and left fusiform gyrus, and increased hippocampal FC with the bilateral middle frontal gyrus, insula, Rolandic operculum, left inferior frontal gyrus (opercular/orbital/triangular parts), and right postcentral/supramarginal/superior temporal gyri. Second, left ventricular end-diastolic diameter (LVDd) was positively associated with GM volume in the left middle temporal gyrus (MTG.L, *p* = 0.016), right inferior temporal gyrus (*p* = 0.006), and left cuneus (*p* = 0.026); MTG.L GM volume correlated positively with Auditory Verbal Learning Test (AVLT)-Recall scores (*p* = 0.044), while hippocampal FC with the right postcentral gyrus correlated negatively with both inflammatory markers (PCT, *p* = 0.010) and AVLT-Delayed Recall (20 min) scores (*p* = 0.013). Third, post catheter ablation (post-CA), AF patients exhibited increased hippocampal FC with the right middle frontal gyrus, right midcingulate cortex, and left superior frontal gyrus, and decreased FC with the right lingual gyrus and calcarine cortex.

**Conclusion:**

Cardiac structural parameters (LVDd) associate with memory-related brain atrophy, whereas blood-borne inflammatory markers link to hippocampal memory network dysregulation—two distinct pathways driving AF-related memory impairment. These findings clarify the dual-pathway regulatory mechanism of the heart-brain axis, offering novel insights into AF-associated cognitive dysfunction and potential CA-mediated memory recovery.

## 1 Introduction

AF—the most prevalent sustained cardiac arrhythmia worldwide (Nielsen et al., 2022)—imposes burdens extending beyond cardiovascular complications: accumulating clinical evidence reveals that 22–51% of patients with AF experience persistent memory impairment (Knecht et al., 2008; Salvadori et al., 2023; Wang et al., 2021). This subclinical cognitive decline not only impairs quality of life but also associates with long-term adverse outcomes, including elevated hospitalization rates, disability rates, and mortality (Bodagh et al., 2022; Madhavan et al., 2018). However, AF is increasingly recognized as a risk factor for memory impairment, but the underlying mechanisms (especially via the heart-brain axis) and whether CA can reverse this impairment remain unclear.

Mounting evidence indicates a significant link between AF diagnosis and cognitive decline (Tang et al., 2023; Wueest et al., 2023). Several investigations have further established that AF patients with cognitive impairment exhibit marked increases in cardiac structural parameters, such as left atrial diameter (LAD), atrial maximum volume index (LAVI), ventricular end-diastolic diameter (LVEDD), ventricular end-systolic diameter (LVESD), and ventricular ejection fraction (LVEF) (Kresge et al., 2020; Krittayaphong et al., 2022; Vos et al., 2022) —parameters closely tied to cognitive functions, particularly memory (Liao and Hou, 2024). Additionally, neuroimaging studies have documented both structural and functional brain abnormalities in AF patients (Silva et al., 2019), with reduced hippocampal GM volume and concurrent memory impairment observed in this population (Knecht et al., 2008; Silva et al., 2019). These findings have preliminarily connected cardiac function to aberrant brain structure/function and cognitive decline (van der Velpen et al., 2017). Nevertheless, the specific pathway by which cardiac function influences cognitive decline via brain structure and function remains unclear. Notably, it is also unknown whether cardiac functional and structural indicators drive memory decline through the same mechanism.

CA is commonly acknowledged as a safe and efficacious first-line intervention for AF and left-sided atrial tachycardia, having demonstrated effectiveness in restoring sinus rhythm and reversing cardiac structural remodeling (Maslova et al., 2024). While CA’s cardiovascular benefits are well-documented, its impact on the hippocampal memory network—a key upstream mediator of AF-related memory impairment—has not been systematically explored. Critical gaps remain: it is unknown whether CA-induced improvements in cardiac structure translate to restored hippocampal FC and memory function, nor whether baseline cardiac structural abnormalities predict the severity of hippocampal network disruption in AF patients.

Against this background, the present study aimed to address three key objectives: first, to evaluate whether AF patients awaiting CA exhibit abnormalities in brain structure (GM), brain function (hippocampal FC), and memory function; second, to investigate how AF-induced changes in cardiac function (structural and functional indicators) relate to GM atrophy, hippocampal memory-associated FC networks, memory performance, and inflammatory cytokines; third, to determine whether CA can enhance the hippocampal memory-related FC network in these patients. We hypothesized that: (a) AF patients awaiting CA would exhibit memory-related atrophy of brain structures and aberrant hippocampal FC; (b) cardiac structural parameters would correlate with memory-related brain structural atrophy, whereas blood-borne inflammatory markers would associate with alterations in hippocampal memory networks; (c) CA-mediated improvements in the hippocampal memory-related network would be accompanied by recovery of memory function post-intervention. Consequently, this study may provide empirical evidence to validate CA as a promising intervention for mitigating brain dysfunction and memory impairment in AF patients, further assisting clinicians in optimizing patient selection for CA and designing targeted post-procedural brain function monitoring strategies to improve long-term patient prognosis.

## 2 Materials and methods

### 2.1 Participants

This study received ethical approval from the Medical Ethics Committee of Nanjing Drum Tower Hospital and all participants provided written informed consent before being enrolled in the study.

The initial cohort included 83 individuals who met eligibility criteria and completed baseline measurements: 45 CN subjects and 38 AF patients scheduled for preoperative CA. AF patients were consecutively recruited via non-selective enrollment between August 2024 and August 2025 at the aforementioned hospital, having been referred for CA as treatment following initial AF ablation. Community-based advertisements, word-of-mouth referrals, and suggestions from healthcare professionals were employed to recruit CN subjects. The CN and AF groups were fully matched for age, with a mean age of 60.04 ± 4.06 years in the CN group and 62.21 ± 9.09 years in the AF group, and no statistically significant differences were observed between the two groups. A difference was noted in the gender ratio: the CN group included 12 males and 33 females, while the AF group consisted of 24 males and 14 females. In the *post-hoc* analysis, gender will be treated as a covariate to adjust for its potential effect.

AF inclusion criteria: AF duration ≥ 6 months, paroxysmal/persistent AF (excluding permanent AF); no history of pharmacological cardioversion within 3 months; no comorbidities including diabetes, moderate-to-severe sleep apnea, anxiety/depression, or general anesthesia surgery within 6 months. Both groups were subject to the same exclusion criteria, which included: (1) substance dependence (on drugs or alcohol) or tobacco use disorder; (2) left-handedness; (3) central nervous system diseases or mental disturbances; (4) chronic illnesses associated with encephalopathy induction; (5) a history of head trauma or craniocerebral operations; (6) severe visual deficits or communication difficulties; and (7) contraindications to magnetic resonance (MR) imaging.

The exclusion methods for Alzheimer’s disease (AD) and vascular cognitive impairment as follows. All AF patients underwent a comprehensive neurocognitive assessment: (1) Mini-Mental State Examination (MMSE) ≥ 27 (to exclude moderate-to-severe cognitive impairment); (2) Clinical Dementia Rating (CDR) = 0 (to exclude AD); (3) MRI examination to exclude cerebral infarction, brain hemorrhage, or other structural lesions (to rule out vascular cognitive impairment); (4) No history of AD or vascular cognitive impairment in medical records. We confirm that all 38 AF patients met these exclusion criteria.

Of the 38 AF patients and 45 CN subjects with usable baseline data, 2 AF patients were excluded post-functional magnetic resonance imaging (fMRI) acquisition due to inadequate image quality. For CN subjects, 3 were excluded for excessive motion [root mean square (RMS) translation > 3 mm, rotation > 3° (Power et al., 2012), or mean framewise displacement (FD) > 0.5 mm] (Yan et al., 2013). This resulted in 36 AF patients and 42 CN subjects for the final baseline analysis.

For postoperative follow-up of AF patients: 4 withdrew after declining CA; 1 was excluded due to poor postoperative fMRI quality; 4 due to excessive fMRI motion (RMS translation > 3 mm, rotation > 3°, or mean FD > 0.5 mm); and 13 for failing to complete postoperative fMRI. Ultimately, 14 AF patients with matched preoperative and postoperative data were included in the final paired analysis.

Neurocognitive assessments, blood sample collection, structural MRI (sMRI), and fMRI were conducted on the same day preoperatively for AF patients and at baseline for CN subjects. These evaluations were repeated for AF patients on postoperative day 7.

### 2.2 Standard clinical protocols of CA

The 38 AF patients scheduled for CA underwent the procedure per standard clinical guidelines (Miyamoto et al., 2023; Miyamoto et al., 2022). Preoperatively, comprehensive evaluations (electrocardiography, echocardiography, medical history review) confirmed AF diagnosis, assessed cardiac structure/function, and ruled out procedural contraindications. Intraoperatively, under fluoroscopic and intracardiac electrogram guidance, a transvenous technique advanced ablation catheters to target atrial tissues (e.g., pulmonary vein ostia, left atrial posterior wall). Radiofrequency or cryoablation energy created discrete lesions to isolate abnormal electrical foci and restore normal rhythm. Postoperatively, patients were monitored for immediate complications (e.g., bleeding, arrhythmia recurrence) via continuous electrocardiographic monitoring and routine blood tests, with follow-up planned to evaluate long-term rhythm control.

### 2.3 Memory assessments

Neuropsychological assessments followed protocols detailed in our prior work (Chen et al., 2019a; Chen et al., 2016; Chen et al., 2015; Chen et al., 2019b; Xue et al., 2019) and evaluated episodic memory at two time points: pre-CA and postoperative day 7. The assessment battery included standardized tests: the Mini-Mental State Examination (MMSE) and the Auditory Verbal Learning Test (AVLT)—with subtests for immediate recall (AVLT-IR), 5-min delayed recall (AVLT-5 min-DR), 20-min delayed recall (AVLT-20 min-DR), and recognition (AVLT-Rec).

### 2.4 Blood biomarker determinations and Cardiac function assessments

Blood biomarker and cardiac function evaluations were performed pre-CA and on postoperative day 7 for all AF patients. All analyses used standardized laboratory protocols and validated methods to ensure accuracy and reproducibility.

For blood analysis: Venous samples were collected and immediately centrifuged to isolate serum (stored appropriately until testing). Assessed biomarkers included two categories:

Coagulation indices: Prothrombin time (PT, reflecting extrinsic/common coagulation pathways), prothrombin activity (PTA, indicating hepatic synthetic function and coagulation factor activity, complementary to PT), activated partial thromboplastin time (APTT, evaluating intrinsic/common coagulation pathways), and thrombin time (TT, measuring fibrinogen-to-fibrin conversion in late coagulation).

Inflammatory cytokines: Interleukin-6 (IL-6, a key pro-inflammatory cytokine regulating acute inflammation and immune activation) and procalcitonin (PCT, a sensitive/specific biomarker for bacterial infection and inflammatory severity, unrelated to cardiac parameters).

Cardiac function was assessed via cardiac ultrasonography, measuring parameters for comprehensive cardiac health evaluation: heart rate (HR), corrected QT interval (QTc, reflecting cardiac electrical activity and arrhythmia risk), left atrial diameter (LAD, an indicator of left atrial remodeling linked to AF progression), left ventricular end-diastolic dimension (LVDd, measuring ventricular chamber size at end-diastole to assess dilation), and left ventricular ejection fraction (LVEF—a critical metric of left ventricular systolic function, calculated as the percentage of blood ejected per contraction). All ultrasonographic measurements followed standard clinical guidelines.

### 2.5 MRI data acquisition

All MRI data were acquired using a United Imaging uMPR 790 scanner, including high-resolution 3D T1-weighted imaging and BOLD-MRI (implemented via gradient echo-planar imaging with its specific parameters), with detailed content of these acquisitions available in Supplementary methods 1.

### 2.6 fMRI preprocessing

The preprocessing steps for all fMRI data were performed following the procedure described in our published paper (Yao et al., 2025), including discarding the first 10 volumes, correcting slice timing and head motion, excluding participants with excessive head motion, co-registering functional and structural images, normalizing/segmenting via the DARTEL algorithm (resampling voxel size, 3 × 3 × 3 mmł), conducting spatial smoothing, performing covariate regression, applying temporal band-pass filtering (0.01–0.10 Hz), and using the DARTEL-generated group GM mask for further analyses; detailed content is available in Supplementary methods 2.

### 2.7 Hippocampal FC analyses

We used seed-based FC analysis in a cohort-specific fashion, with the left hippocampus (defined by the AAL3v1 atlas) as the seed region, where distinct analyses were conducted for each group. For every individual in the study, the average time series of all voxels in the left hippocampus was extracted to act as the reference time series. Next, voxel-level cross-correlation was carried out: This entailed calculating correlations between the mean time series of the hippocampal seed region and the time series of each individual voxel in other brain regions, with all computations confined to the GM mask unique to each group. Finally, Fisher’s z-transformation was applied to the correlation coefficients to improve their normality—a key step for subsequent statistical analyses.

### 2.8 Voxel-based morphometry analysis

Voxel-based morphometry (VBM) analysis was done via DPABI (built on MATLAB R2023a), including segmenting 3D T1-weighted images into GM/white matter (WM)/cerebrospinal Fluid (CSF) via DARTEL, normalizing GM to MNI space, modulating for volume correction, and smoothing with an 8 mm FWHM Gaussian kernel; details are in Supplementary methods 3.

### 2.9 Statistical analysis

#### 2.9.1 Demographics and neuropsychological data

For continuous variables, data are reported as mean (standard deviation), while categorical variables (e.g., gender) are presented as frequencies. Independent samples *t*-tests were employed to compare continuous variables, and Chi-square tests were used for the comparison of categorical variables.

#### 2.9.2 Comparisons of gray matter and hippocampal functional network connectivity between the CN group and the AF group

At baseline, two-sample *t*-tests were utilized to evaluate differences in GM and hippocampal FCs within the GM mask between the two groups, with adjustments for age, sex, and head motion. Regions with significant voxel numbers ≥ 100 (2,700 mm^3^) with TFCE-FWE-corrected *P* < 0.01 were presented and defined from the regions of interest (ROIs) in AAL3v1 atlas (Rolls et al., 2020).

#### 2.9.3 Comparisons of hippocampal functional network connectivity between patients with pre-operative and post-operative AF undergoing CA

Paired *t*-test was used to assess the differences in the hippocampal FCs within GM mask between the preAF-CA group and the postAF-CA group. All results were controlled for age, sex, and head motion at a threshold of *p* < 0.05 using TFCE correction with cluster size > 30 voxels (810 mm^3^).

#### 2.9.4 Relationships among altered GM, hippocampal FC, memory, and clinical features

Bivariate correlation analyses were performed to elucidate the relationships among altered GM, FC, four coagulation indices (PTA, APTT, TT, and PT), inflammation levels (IL-6 and PCT), cardiac function (HR, QTc, LVDd, LAD, and LVEF), and memory performance in AF patients.

## 3 Results

### 3.1 Demographic and neuropsychological characteristics

As shown in Table 1, AF displayed lower MMSE, AVLT-IM, and AVLT-Rec scores than those in CN (*P* < 0.05). No other significant differences were observed across the remaining tests when comparing the two groups (*P* > 0.05).

**TABLE 1:** Demographic, *clinical*, and *cognitive characteristic*s of the AF and CN *groups.*

Items	CN group	AF group	*P-*values
	*n* = 45	*n* = 38	
**Demographic characteristics**			
Age (years)	62.04(4.06)	62.21(9.09)	0.917
Gender (male/female)	12/33	24/14	0.001***
**Coagulation four indices**			
PT	–	11.44(1.00)	–
PTA	–	96.86(19.74)	–
APTT	–	28.02(3.06)	–
TT	–	18.03(0.82)	–
**Inflammatory cytokines**			
PCT (ng/mL)	–	0.03(0.01)	–
IL-6 (pg/mL)	–	4.02(3.61)	–
**Cardiac function**			
HR	–	77.95(17.15)	–
QTC	–	437.67(30.42)	–
LAD (cm)	–	4.22(0.60)	–
LVDd (cm)	–	4.96(0.40)	–
LVEF (%)	–	58.08(5.86)	–
**Cognitive assessment**			
MMSE	29.02(0.89)	26.53(3.84)	0.001***
AVLT-IM	16.91(3.95)	12.23(4.54)	<0.001***
AVLT-DR-5 min	5.76(2.07)	4.80(3.07)	0.111
AVLT-DR-20 min	5.20 (2.25)	3.97(3.46)	0.091
AVLT-Rec	21.73(1.47)	18.33(5.55)	0.003**

Data are presented as the mean (standard deviation). **p < 0.01, ***p < 0.001. CN, healthy controls; AF, atrial fibrillation; MMSE, mini-mental state examination; AVLT-IM, auditory verbal learning test—immediate recall; AVLT-DR-5 min, auditory verbal learning Test—5-min delayed recall; AVLT-20 min-DR, auditory verbal learning test—20-min delayed recall; AVLT-Rec, auditory verbal learning test—recognition; TMT-A, trail making test-A; verFluency, verbal fluency test; CDT, clock drawing test; TMT-B, trail making test-B; PT, prothrombin Time; PTA, prothrombin Activity; APTT, activated partial thromboplastin Time; TT, thrombin Time; PCT, procalcitonin; IL-6, Interleukin-6; HR, heart rate; QTC, Corrected QT Interval; LAD, left atrial diameter; LVDd, left ventricular end-diastolic dimension; LVEF, left ventricular ejection fraction. Missing Data in AF: 10 missing values per indicator (28/38 valid) for AVLT-IM, AVLT-DR-5 min, AVLT-DR-20 min, AVLT-Rec, TMT-A, verFluency, CDT 30, TMT-B; 2 missing values per indicator (36/38 valid) for PT, INR, PTA, APTT, TT; 7 missing values per indicator (31/38 valid) for PCT, IL-6; 17 missing values per indicator (21/38 valid) for HR, QTC; and 9 missing values per indicator (29/38 valid) for LAD, LVDd, EF.

### 3.2 Comparisons of gray matter between the AF group and CN group

**FIGURE 1:**
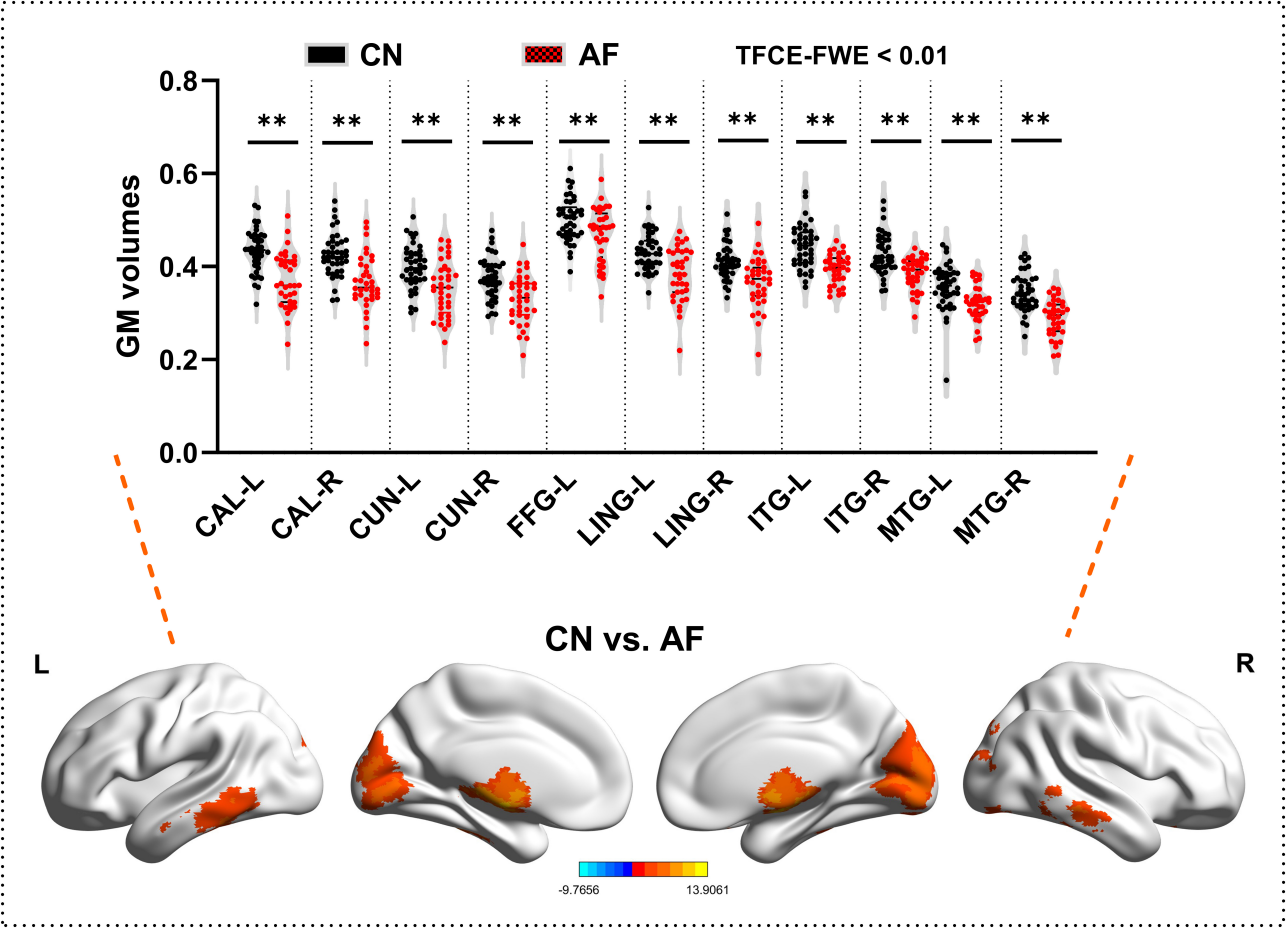
Grey matter differences between the AF group and CN group at baseline. The results from two-sample *t*-test are displayed using TFCE-FWE-corrected *p* < 0.01 and cluster extent *k* > 100 voxels (2,700 mm^3^). Results are presented after controlling for age and sex. **p < 0.01. L, left hemisphere; R, right hemisphere; AF, Atrial fibrillation; CN, healthy controls; GM, Gray matter; MTG, middle temporal gyrus; ITG, inferior temporal gyrus; FGG, fusiform gyrus; LING, lingual gyrus; CAL, calcarine fissure and surrounding cortex; CUN, cuneus.

As depicted in Figure 1 and Table 2, AF group exhibited significantly decreased GM in bilateral CAL, CUN, LING, ITG, MTG, and left FFG that CN group (TFCE-FWE-corrected *P* < 0.01 and cluster extent *k* > 100 voxels).

**TABLE 2:** Hippocampal functional connectivity differences between the AF group and CN group.

Brain regions	L/R	MNI			*T-*values	Cluster size (mm^3^)
		x	y	z		
IFGoperc	L	–51	18	12	–4textbf.6154	3,996
IFGorb	L	–45	15	–9	–4.3943	2,808
IFGtriang	L	–48	18	15	–4.5016	10,368
MFG	L	–30	39	21	–4.1712	2,700
MFG	R	36	39	21	–5.3584	3,294
Insula	L	–36	–6	–15	–5.9262	9,477
Insula	R	39	9	–15	–3.973	3,861
PoCG	R	65	–15	33	–4.9299	3,942
ROL	L	–42	0	18	–4.6859	2,889
ROL	R	57	–18	18	–3.8106	4,536
SMG	R	63	–42	24	–5.8874	4,563
STG	R	60	–42	21	–6.2954	5,211

Regions with significant voxel numbers ≥100 with TFCE-FWE correction were presented and defined from the regions of interest (ROIs) in AAL3v1 atlas (Rolls et al., 2020). MNI, montreal neurological institute; L, left hemisphere; R, right hemisphere. AF, Atrial fibrillation; CN, healthy controls. IFGoperc, Inferior frontal gyrus, opercular part; IFGtriang, Inferior frontal gyrus, triangular part; IFGorb, IFG pars orbitalis; MFG, middle frontal gyrus; PoCG, postcentral gyrus; ROL, rolandic operculum; SMG, supramarginal gyrus; STG, superior temporal gyrus. Results are presented after controlling for age, sex, and head motion.

### 3.3 Comparisons of hippocampal functional connectivity network between the AF group and CN group

**FIGURE 2:**
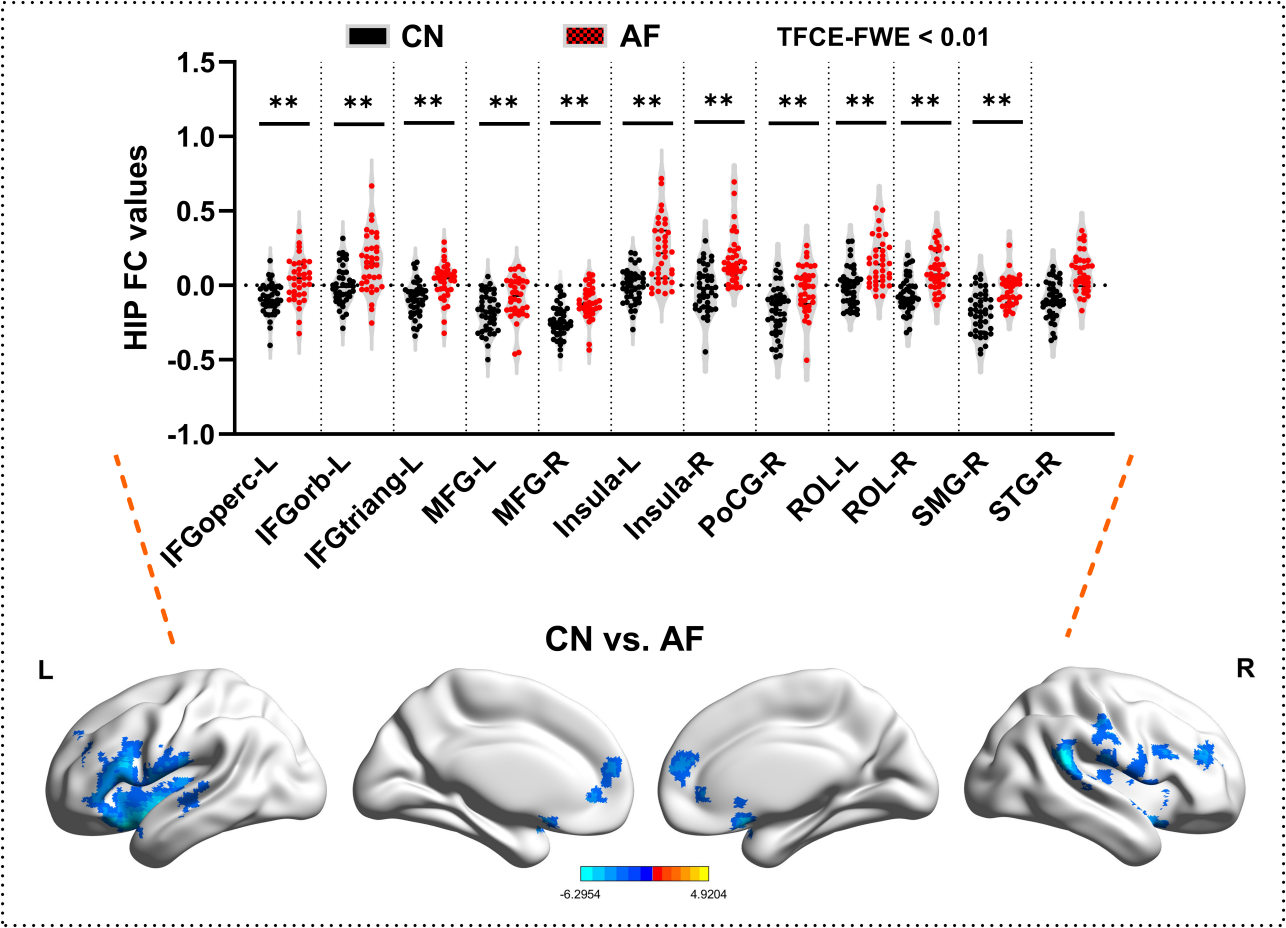
Hippocampal functional connectivity differences between the AF group and CN group at baseline. The results from two-sample *t*-test are displayed using TFCE-FWE-corrected *p* < 0.01 and cluster extent *k* > 100 voxels (2,700 mm^3^). Results are presented after controlling for age and sex. **p < 0.01. L, left hemisphere; R, right hemisphere; AF, Atrial fibrillation; CN, healthy controls. IFGoperc, Inferior frontal gyrus, opercular part; IFGtriang, Inferior frontal gyrus, triangular part; IFGorb, IFG pars orbitalis; MFG, middle frontal gyrus; PoCG, postcentral gyrus; ROL, rolandic operculum; SMG, supramarginal gyrus; STG, superior temporal gyrus.

As depicted in Figure 2 and Table 3, AF group exhibited significantly increased FC in bilateral MFG, bilateral Insula, bilateral ROL, left IFGoperc, left IFGorb, left IFGtriang, right PoCG, right SMG, and right STG (TFCE-FWE-corrected *P* < 0.01 and cluster extent *k* > 100 voxels).

**TABLE 3:** Grey matter differences between the AF group and CN group.

Brain regions	L/R	MNI			*T-*values	Cluster size (mm^3^)
		x	y	Z		
CAL	L	6	–93	0	6.1646	7,317
CAL	R	6	–93	12	6.5052	4,995
CUN	L	3	–90	15	5.5599	3,942
CUN	R	9	–90	18	7.0319	4,077
FFG	L	–45	–39	–21	5.7071	2,754
LING	L	–6	–78	3	5.7569	3,591
LING	R	6	–87	–6	5.6757	3,483
ITG	L	–48	–42	–24	7.5579	8,856
ITG	R	66	–24	–21	5.9103	6,561
MTG	L	–48	–33	–18	4.9134	5,589
MTG	R	69	–15	–18	10.7515	7,992

Regions with significant voxel numbers ≥100 with TFCE-FWE correction were presented and defined from the regions of interest (ROIs) in AAL3v1 atlas (Rolls et al., 2020). MNI, montreal neurological institute; L, left hemisphere; R, right hemisphere. AF, Atrial fibrillation; CN, healthy controls. MTG, middle temporal gyrus; ITG, inferior temporal gyrus; FFG, fusiform gyrus; LING, lingual gyrus; CAL, calcarine fissure and surrounding cortex; CUN, cuneus. Results are presented after controlling for age and sex.

### 3.4 Relationships among altered GM, hippocampal FC, memory, and clinical features

**FIGURE 3:**
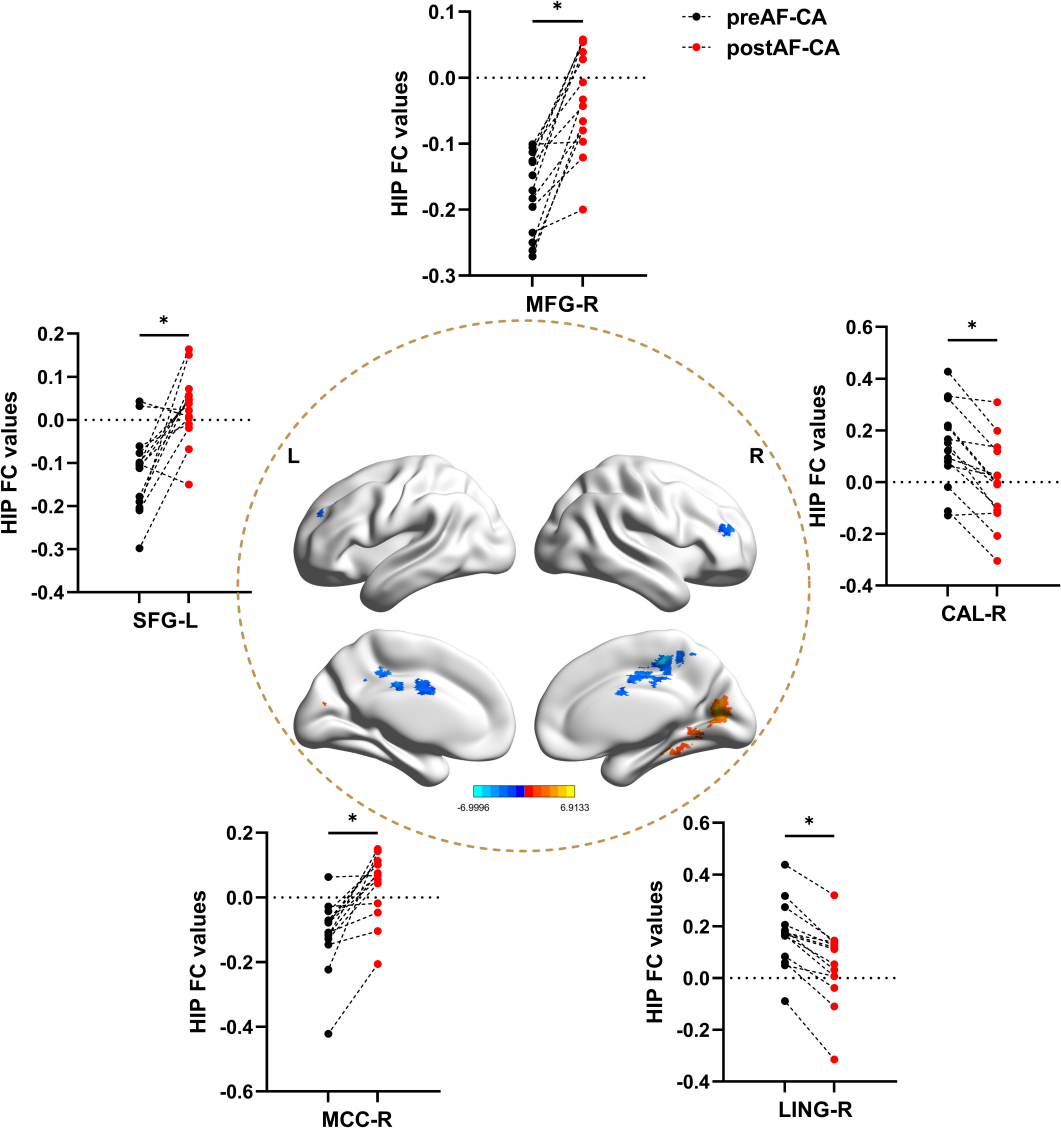
Hippocampal functional connectivity differences between patients with pre-operative and post-operative atrial fibrillation undergoing catheter ablation. All results were controlled for age, sex, and head motion at a threshold of *p* < 0.05 using TFCE correction with cluster size > 30 voxels (810 mm^3^). *p < 0.05. L, left hemisphere; R, right hemisphere; preAF-CA, patients with pre-operative atrial fibrillation undergoing catheter ablation; postAF-CA, patients with post-operative atrial fibrillation undergoing catheter ablation; MFG, middle frontal gyrus; SFG, superior frontal gyrus; CAL, Calcarine gyrus; MCC, Middle cingulate and paracingulate gyrus; LING, Lingual gyrus.

This study inivestigated the relationships among altered GM, hippocampal FC, memory, and clinical features (cardiac indicator: HR, QTc, LAD, LVDd, and LVEF; inflammation levels: IL-6 and PCT). As shown in Figure 3, LVDd was significantly positively correlated with GM in CUN.L (*r* = 0.427, *p* = 0.026), ITG.R (*r* = 0.519, *p* = 0.006), and MTG.L (*r* = 0.460, *p* = 0.016), and altered GM in MTG.L was significantly positively correlated with AVLT-Rec score (*r* = 0.398, *p* = 0.044). Furthermore, altered FC between PoCG.R and hippocampus was significantly negatively correlated with the inflammatory indicator (PCT, *r* = –0.477, *p* = 0.010) and AVLT-DR-20 min score (*r* = –0.482, *p* = 0.013).

### 3.5 Hippocampal FC changes between pre-operative and post-operative atrial fibrillation patients undergoing catheter ablation

**FIGURE 4:**
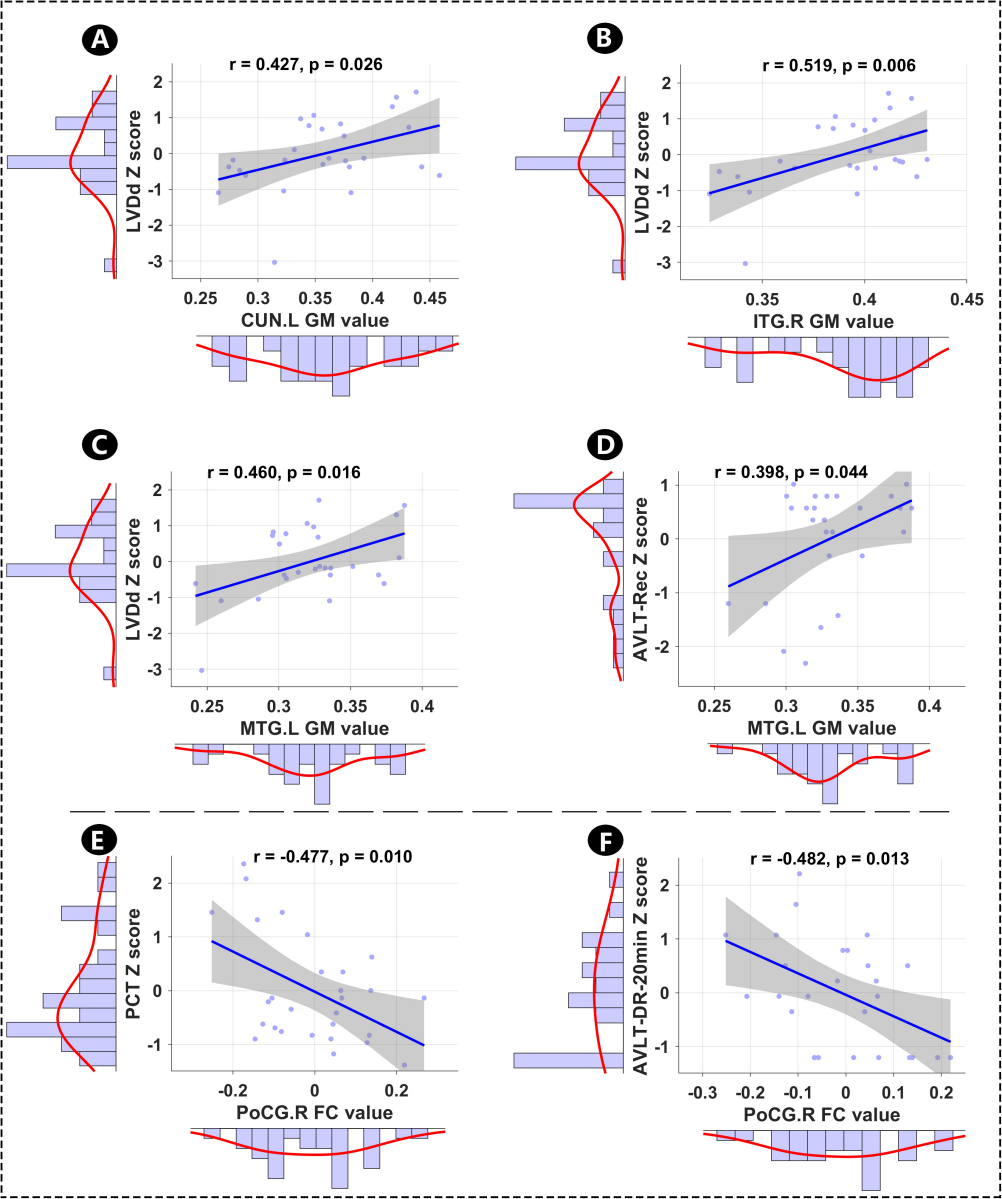
Relationships among mean altered GM, hippocampal FC, memory, and clinical features. **(A–D)** Relationships between altered GM and AVLT-Rec, and between altered GM and LVDd. **(E,F)** Relationships between hippocampal FC and PCT, and between hippocampal FC and AVLT-20 min-DR. LVDd, left ventricular end-diastolic dimension; L, left hemisphere; R, right hemisphere; CUN, cuneus; ITG, inferior temporal gyrus; PoCG, postcentral gyrus; MTG, middle temporal gyrus; PCT, procalcitonin; AVLT-Rec, auditory verbal learning test—recognition; AVLT-20 min-DR, auditory verbal learning test—20-min delayed recall; GM, gray matter; FC, functional conn.

As shown in Figure 4 and Table 4, when compared with the pre-operative AF group, the post-operative AF group exhibited increased FC between the MFG.R, SFG.L, MCC.R and the hippocampus; decreased FC between the LING.R, CAL.R, and the hippocampus.

**TABLE 4:** Hippocampal functional connectivity differences between the preAF-CA group and postAF-CA group.

Brain regions	L/R	MNI			*T-*values	Cluster size (mm^3^)
		x	y	Z		
MFG	R	42	51	21	–5.7123	8,262
SFG	L	–24	42	48	–3.8843	3,078
CAL	R	9	–72	12	6.9133	6,318
MCC	R	9	–30	51	–6.9996	10,800
LING	R	30	–33	–18	5.1987	2,916

Regions with significant voxel numbers ≥30 with TFCE correction were presented and defined from the regions of interest (ROIs) in AAL3v1 atlas (Rolls et al., 2020). MNI, montreal neurological institute; L, left hemisphere; R, right hemisphere; preAF-CA, patients with pre-operative atrial fibrillation undergoing catheter ablation; postAF-CA, patients with post-operative atrial fibrillation undergoing catheter ablation. MFG, middle frontal gyrus; SFG, superior frontal gyrus; CAL, Calcarine gyrus; MCC, Middle cingulate & paracingulate gyrus; LING, Lingual gyrus.

## 4 Discussion

AF has long been implicated in cognitive decline, yet the specific brain structural and functional correlates linking cardiac dysfunction to memory impairment remain incompletely characterized—especially in patients undergoing CA, a first-line intervention for AF. To our knowledge, the present study was the first to answer questions about the specific pathway by which cardiac function influences memory decline via brain structure and function. The present study addresses this gap by integrating multimodal neuroimaging, clinical cardiac assessments, and memory testing, yielding three core findings that advance understanding of the heart-brain axis in AF. Furthermore, CA-mediated improvements in the hippocampal memory-related network would be accompanied by recovery of memory function post-intervention. Consequently, this study may provide empirical evidence to validate CA as a promising intervention for mitigating brain dysfunction and memory impairment in AF patients, further assisting clinicians in optimizing patient selection for CA and designing targeted post-procedural brain function monitoring strategies to improve long-term patient prognosis.

Several methodological and clinical strengths enhance the rigor and relevance of our findings. Methodologically: (1) We integrated structural (voxel-based morphometry) and functional (hippocampal FC) MRI—alongside comprehensive clinical assessments (memory tests, cardiac function metrics, blood biomarkers)—to establish multimodal links between cardiac structure, brain integrity, and memory. (2) Pre- and post-CA assessments of AF patients, paired with a CN group, enabled longitudinal evaluation of CA’s impact on the brain and controlled comparison of AF-related abnormalities. (3) Strict correlation analyses isolated the specific relationships between cardiac LVDd, inflammatory, and neuroimaging markers with memory, avoiding confounds from univariate analyses. Clinically: (1) We identified region-specific GM and FC alterations (e.g., left middle temporal gyrus, right postcentral gyrus-hippocampal FC) that serve as potential neuroimaging biomarkers for AF-related memory impairment, moving beyond broad “brain dysfunction” frameworks. (2) By linking CA to FC normalization, we provide preliminary evidence that CA may target not only cardiac but also brain-based drivers of memory decline, supporting its expanded clinical value. (3) The rationale for choosing postoperative day 7 was because our previous studies (Yao et al., 2025) have shown that CA-induced acute inflammation and cardiac function fluctuations stabilize by day 7, avoiding interference from short-term physiological disturbances on brain structure/function assessments.

Our findings showed that relative to CN, AF patients awaiting CA exhibited robust memory impairment accompanied by region-specific GM volume reductions and hippocampal FC alterations. The GM loss observed in the bilateral calcarine cortex, cuneus, lingual gyrus, inferior temporal gyrus, middle temporal gyrus, and left fusiform gyrus is particularly notable, as these regions collectively support visual-spatial information (Han et al., 2025), semantic and spatial Memory (Gonzalez Alam et al., 2025), and episodic memory (Chen et al., 2022; Chen et al., 2016)—domains consistently disrupted in AF-related cognitive decline (van der Velpen et al., 2017). Complementing these structural changes, increased hippocampal FC with the bilateral middle frontal gyrus, insula, Rolandic operculum, left inferior frontal gyrus subregions, and right postcentral/supramarginal/superior temporal gyri suggests a compensatory or pathological reconfiguration of memory-related networks. The insula and middle frontal gyrus, for instance, are critical for interoceptive processing and executive control (Xu et al., 2020); their heightened connectivity with the hippocampus may reflect an adaptive response to cardiac-induced brain dysfunction, though this requires further validation. The possible mechanisms underlying the correlation between cardiac structural changes and brain GM/FC alterations are: (1) Cerebral hypoperfusion: Enlarged left atrium (a key cardiac structural change) reduces cardiac output, leading to decreased cerebral blood flow to the hippocampus and frontal lobe—this hypoperfusion impairs neuronal metabolism, resulting in GM volume loss and FC reduction (van der Velpen et al., 2017). (2) Inflammatory cascade: Cardiac structural remodeling (e.g., myocardial hypertrophy)

triggers systemic inflammation, which crosses the blood-brain barrier to activate microglia, damaging hippocampal neurons and disrupting FC (Silva et al., 2019; Yao et al., 2025).

Our most fascinating finding was that our correlation analyses uncovered distinct pathways linking cardiac structure, inflammation, and memory. The positive association between LVDd—a key marker of cardiac structural remodeling—and GM volume in the left cuneus, right inferior temporal gyrus, and left middle temporal gyrus aligns with the hypothesis that cardiac dilatation may modulate cerebral perfusion or vascular integrity, thereby influencing GM integrity in memory-relevant regions (van der Velpen et al., 2017). Notably, the positive correlation between GM volume of left middle temporal gyrus and AVLT-Recall scores directly links structural preservation in this region to better memory performance, reinforcing the role of middle temporal gyrus in verbal memory encoding (Liao and Hou, 2024; Tendolkar et al., 2012). In contrast, the negative correlations between blood-borne inflammatory markers, right postcentral gyrus -hippocampal FC, and AVLT- DR-20 min scores highlight a separate inflammatory pathway: systemic inflammation, a well-documented comorbidity of AF, may disrupt hippocampal network coherence (e.g., by impairing synaptic plasticity) and thereby exacerbate memory deficits—extending prior work that linked inflammation to AF-related cognitive impairment (Hu et al., 2023; Yao et al., 2025). Therefore, these findings shed light on the two distinct pathways of regulatory mechanism of the heart-brain axis underlying memory impairment in AF patients.

Another significant result showed that CA-induced changes in hippocampal FC further support the intervention’s potential to modulate the heart-brain axis. Postoperatively, AF patients exhibited increased hippocampal FC with the right middle frontal gyrus, left superior frontal gyrus, and right midcingulate cortex—regions involved in cognitive control and memory consolidation (Vogt et al., 2013)—and decreased FC with the right lingual gyrus and right calcarine cortex. These changes may reflect CA-mediated improvements in cardiac function (e.g., restored sinus rhythm, reduced cardiac remodeling) that alleviate cerebral vascular stress, thereby normalizing hippocampal network dynamics. Short-term improvement mechanism: CA restores sinus rhythm, reducing left atrial pressure and improving cardiac output—this enhances cerebral perfusion to the hippocampus and memory-related regions, reversing acute hypoperfusion-induced cognitive impairment. Additionally, CA reduces cardiac structural remodeling-induced inflammation (decreased CRP, IL-6), alleviating neuroinflammation-mediated brain dysfunction. A follow-up study showed that AF patients who maintained sinus rhythm after CA had better cognitive function (MMSE score) than those with AF recurrence, supporting CA’s long-term cognitive benefits (Maslova et al., 2024). While the present study only assessed FC at 7 days post-CA, these acute changes lay the groundwork for investigating whether longer-term CA benefits translate to sustained memory recovery.

Despite these strengths, several limitations should be acknowledged. First, the sample size of AF patients (*n* = 38) is relatively small, particularly for detecting subtle post-CA changes or subgroup effects. While the inclusion of 45 CN strengthens group comparisons, larger cohorts are needed to validate the generalizability of our correlation findings (e.g., LVDd-GM associations). Second, we acknowledge that 7-day short-term follow-up cannot confirm the sustainability of changes, and note that we are currently conducting a 6-month follow-up study to verify whether hippocampal FC and memory improvements persist. Long-term follow-up will help clarify CAmpal FC-term role in modulating the heart-brain axis. Third, we did not account for potential confounders such as comorbid vascular disease, medication use (e.g., anticoagulants), or baseline cognitive status, which may independently influence GM, FC, or memory. Fourth, we did not assess CA efficacy (e.g., maintenance of sinus rhythm) post-intervention; linking CA’s cardiac success to brain/memory outcomes would clarify whether FC changes are directly mediated by improved cardiac function. Finally, this study did not separately evaluate the short-term impact of the CA procedure itself (e.g., procedural stress) and anesthesia methods (e.g., propofol) on cognitive function. Previous studies have shown that short-term cognitive dysfunction (postoperative cognitive dysfunction) can occur within 1 week after anesthesia/surgery, which may partially overlap with the cognitive changes we observed. Future studies should include a control group (e.g., AF patients undergoing non-invasive treatment) to isolate the specific effect of CA on cognition.

## 5 Conclusion

This study delineates two distinct pathways through which AF contributes to memory impairment: cardiac structural remodeling (via LVDd) associated with GM atrophy in memory-relevant regions, and systemic inflammation associated with hippocampal FC disruption. Additionally, we provide preliminary evidence that CA may normalize hippocampal FC, suggesting it could mitigate brain-based memory deficits. Clinically, these findings offer three key insights: (1) Neuroimaging markers may help identify AF patients at high risk of memory impairment, guiding targeted interventions. (2) CA’s potential to modulate hippocampal FC supports its evaluation as a “dual-benefit” intervention for AF patients with cognitive concerns. (3) The separation of cardiac structural and inflammatory pathways highlights the need for multimodal treatments (e.g., CA plus anti-inflammatory therapy) for AF patients with severe memory impairment. Future work should address our limitations to solidify these conclusions and translate them into clinical practice.

## Data Availability

The original contributions presented in this study are included in this article/Supplementary material, further inquiries can be directed to the corresponding authors.
