# Supplementary material for: Cardiac structure and inflammation drive memory impairment via dual pathways of heart-brain axis dysregulation in atrial fibrillation patients undergoing catheter ablation
Source: Front Neurosci. 2025 Nov 27;19:1725953. doi: 10.3389/fnins.2025.1725953 (PMC12695847; doi:10.3389/fnins.2025.1725953)
Supplement: Supplementary file 1 [file Data_Sheet_1.docx]

**Cardiac structure and inflammation drive memory impairment via dual pathways of heart-brain axis dysregulation in atrial fibrillation patients undergoing catheter ablation**

**Xin Chen^1,2,#^,** **Jie Ni ^3,#^, Yu Wang^2^,** **Dandan Kou^4,5,6^, Danni Ge****^4,5,6^,** **Xunwen Xue^4,5,6^, Yingming Zhao^2^, Ming Li ^4,5,6,^*, Jiu Chen^4,5,6,^*, Biao Xu^1 ,2^***

^1^Department of Cardiology, Nanjing Drum Tower Hospital, Nanjing Drum Tower Hospital Clinical College of Nanjing Medical University, No. 321 Zhongshan Road, Nanjing 210008, China.

^2^Department of Cardiology, Nanjing Drum Tower Hospital, Affiliated Hospital of Medical School, Nanjing University, No. 321 Zhongshan Road, Nanjing 210008, China.

^3^General Medical Department, Nanjing Drum Tower Hospital, the Affiliated Hospital of Nanjing University Medical School, No. 321 Zhongshan Road, Nanjing 210008, China.

^4^Department of Radiology, Nanjing Drum Tower Hospital, Affiliated Hospital of Medical School, Nanjing University, No. 321 Zhongshan Road, Nanjing 210008, China

^5^Institute of Medical Imaging and Artificial Intelligence, Nanjing University, No. 321 Zhongshan Road, Nanjing 210008, China

^6^Medical Imaging Center, Nanjing Drum Tower Hospital, Affiliated Hospital of Nanjing University Medical School, No. 321 Zhongshan Road, Nanjing 210008, China

***Running Title***:

**^#^ Xin Chen** and **Jie Ni** contributed equally to this work (joint first authors).

***Correspondence to**:

**Biao Xu,** Department of Cardiology, Nanjing Drum Tower Hospital, Nanjing Drum Tower Hospital Clinical College of Nanjing Medical University, No. 321 Zhongshan Road, Nanjing 210008, China. E-mail: [xubiao62@nju.edu.cn](mailto:xubiao62@nju.edu.cn)

**Jiu Chen**, Department of Radiology, Nanjing Drum Tower Hospital, Affiliated Hospital of Medical School, Nanjing University, No. 321 Zhongshan Road, Nanjing 210008, China. E-mail: [ericcst@aliyun.com](mailto:ericcst@aliyun.com)

**Ming Li,** General Medical Department, Nanjing Drum Tower Hospital, the Affiliated Hospital of Nanjing University Medical School, No. 321 Zhongshan Road, Nanjing 210008, China. E-mail: lm069393@163.com

**SI methods**

**S.1 MRI data acquisition**

**S.2 fMRI image preprocessing**

**S.3 Voxel-based morphometry analysis**

**SI methods**

**S.1** **MRI data acquisition**

All MRI data were acquired using standard MRI scanning (United Imaging uMPR 790). The MRI session included high-resolution 3D T1-weighted imaging and BOLD-MRI.

The T1-weighted sequence parameters were as follows: repetition time (TR) = 7900 ms, echo time (TE) = 3 ms, field of view (FOV) = 172 mm×155 mm, flip angle (FA) = 10 °, thickness = 0.67 mm, gap = 0.67 mm, acquisition matrix = 256×232, number of excitations (NEX) = 1, bandwidth = 250 Hz. All of the brain data were acquired in the axial plane, yielding 264 continuous slices with an acquired voxel size of 0.67×0.67×0.67mm^3^.

The BOLD-MRI was carried out using gradient echo-planar imaging with the following parameters: TR = 2000 ms, TE = 30 ms，number of slices = 61, thickness = 4 mm, gap = 4 mm, matrix = 64× 70, FA = 90°, FOV = 192 mm×210 mm, voxel size = 3×3×4 mm^3^.

**S.2 fMRI image preprocessing**

All fMRI data were preprocessed using MATLAB2023a (<http://www.mathworks.com/products/matlab/>) and DPABI image processing software (Yan, Wang, Zuo, & Zang, 2016). The image processing procedure was as previously described (Yan, Craddock, Zuo, Zang, & Milham, 2013). The image processing procedure was as follows: the first ten volumes were discarded to reduce the instability of MRI signal. Corrections were performed for the intra-volume acquisition time differences among slices and inter-volume motion effects during the scan (slice timing correction and head motion correction) (Power, Barnes, Snyder, Schlaggar, & Petersen, 2012; Van Dijk, Sabuncu, & Buckner, 2012). Participants with excessive head motion (cumulative translation or rotation > 3.0 mm or 3.0°) were excluded. Individual functional and structural images were co-registered. The Diffeomorphic Anatomical Registration Through Exponentiated Lie Algebra (DARTEL) algorithm was used to normalize and segment the structural images into GM, WM and cerebrospinal fluid (CSF) partitions (Ashburner & Friston, 2009). After realigning, slice timing correction, and co-registration, functional images were normalized by EPI template into MNI space (resampling voxel size, 3 × 3 × 3 mm³) and were then spatially smoothed by a Gaussian kernel of 6 mm^3^ full-width at half maximum (FWHM) to reduce spatial noise. We next used a Friston 24-parameter model (i.e., 6 head motion parameters, 6 head motion parameters one time point before, and the 12 corresponding squared items) to regress out head motion effects from the realigned data (Friston, Williams, Howard, Frackowiak, & Turner, 1996). The WM, CSF, and the global signals as well as the linear trend were also regressed as nuisance covariates (Brady et al., 2019). Framewise displacement (FD) was calculated for all resting state volumes (Power et al., 2012). All volumes with a FD greater than 0.2 mm were regressed out as nuisance covariates (Brady et al., 2019). Any scan with 50% of volumes removed was discarded (Brady et al., 2019). After nuisance covariate regression, temporal band-pass filtering (0.01–0.1 Hz) was applied to reduce the effect of low-frequency drifts and high-frequency physiological noise. Voxels within a group GM mask created by DARTEL were used for further analyses. Forthermore, we computed total intracranial volumes (ITV) based on native GM, WM, and CSF by using in-home MATLAB codes.

**S.3 Voxel-based morphometry analysis**

Voxel-based morphometry (VBM) analysis was performed using the DPABI software, built on the MATLAB R2023a (MathWorks, Natick, MA, USA). Initially, 3D T1-weighted images were segmented into gray matter (GM), white matter (WM), and cerebrospinal fluid (CSF) utilizing the diffeomorphic anatomical registration through exponentiated Lie algebra (DARTEL) algorithm. Subsequently, the GM segments were spatially normalized to the Montreal Neurological Institute (MNI) standard space using DARTEL registrations. Following normalization, the GM images were modulated to correct for volume changes incurred during the normalization process. This modulation step involved multiplying the voxel values by the Jacobian determinants derived from the spatial normalization step. Finally, the modulated and normalized images were smoothed using an 8 mm full width at half maximum (FWHM) Gaussian kernel.

**References**

Ashburner, J., & Friston, K. J. (2009). Computing average shaped tissue probability templates. *Neuroimage, 45*(2), 333-341. doi:10.1016/j.neuroimage.2008.12.008

Brady, R. O., Jr., Gonsalvez, I., Lee, I., Ongur, D., Seidman, L. J., Schmahmann, J. D., . . . Halko, M. A. (2019). Cerebellar-Prefrontal Network Connectivity and Negative Symptoms in Schizophrenia. *Am J Psychiatry*, appiajp201818040429. doi:10.1176/appi.ajp.2018.18040429

Friston, K. J., Williams, S., Howard, R., Frackowiak, R. S., & Turner, R. (1996). Movement-related effects in fMRI time-series. *Magn Reson Med, 35*(3), 346-355.

Power, J. D., Barnes, K. A., Snyder, A. Z., Schlaggar, B. L., & Petersen, S. E. (2012). Spurious but systematic correlations in functional connectivity MRI networks arise from subject motion. *Neuroimage, 59*(3), 2142-2154. doi:10.1016/j.neuroimage.2011.10.018

Van Dijk, K. R., Sabuncu, M. R., & Buckner, R. L. (2012). The influence of head motion on intrinsic functional connectivity MRI. *Neuroimage, 59*(1), 431-438. doi:10.1016/j.neuroimage.2011.07.044

Yan, C. G., Craddock, R. C., Zuo, X. N., Zang, Y. F., & Milham, M. P. (2013). Standardizing the intrinsic brain: towards robust measurement of inter-individual variation in 1000 functional connectomes. *Neuroimage, 80*, 246-262. doi:10.1016/j.neuroimage.2013.04.081

Yan, C. G., Wang, X. D., Zuo, X. N., & Zang, Y. F. (2016). DPABI: Data Processing & Analysis for (Resting-State) Brain Imaging. *Neuroinformatics, 14*(3), 339-351. doi:10.1007/s12021-016-9299-4
